# Anaphylaxis during specific subcutaneous immunotherapy against ragweed allergen -- case record

**DOI:** 10.1186/2045-7022-4-S3-P75

**Published:** 2014-07-18

**Authors:** Besim Prnjavorac, Amina Deljkić, Albina Sinanović, Rifat Sejdinović, Amila Mehedović, Jasna Jukić, Katarina Krajina, Lejla Šaranović, Nedžada Irejiz, Indira Kašibović, Tamer Bego, Maja Malenica, Tanja Dujić, Sabina Semiz, Adlija Čaušević

**Affiliations:** 1 General Hospital Tešanj, Pulmology, Immunology-allergology, Bosnia Herzegowina; 2 Department of Internal Medicine, Gastroenterology-hepatology, Bosnia Herzegowina; 3 General Hospital Tešanj, Department of Anestesiology, Bosnia Herzegowina; 4 University Pittsburgh, Pensilvania, Medical Science, USA; 5 Department of Internal Medicine, Gatroenterology, Bosnia Herzegowina; 6 Department of Internal Medicine, Pulmology, Immunology-allergology, Bosnia Herzegowina; 7 Faculty of Pharmacy, Clinical Biochemistry, Bosnia Herzegowina

## Background

Recommended by Resolution of Immunotherapy, issued by EAACI, Specific immunotherapy (SIT) was established as a mainstream method of treating allergic diseases. SIT produces long term challenges. SIT team should be aware that at first injection of allergen, the immunotherapy may cause a long lasting reaction. Anaphylaxis during SIT is very rare, but it is possible. We've experienced anaphylaxis after four year of SIT, against ragweed allergen.

## Methods

A fifty four year old female went into anaphylactic shock after four years of SIT against ragweed allergen. Before the start of SIT, complete diagnostic procedures had been performed. Intradermal skin tests were performed for standard pallet of contact, inhalation, and nutritional allergens. The female patient tested positive for Ragweed (high positive), fungi (aspergillum fumigates, altenaria), and milk. Total IgE was measured using Enzyme Linked Immunoassay (ELISA).

## Result

After injection of the SIT maintenance dose (0.2 ml of second concentration of allergen, (product of authorized Immunologic Laboratory Production) against ragweed, anaphylaxis developed within five minutes. The first manifestation was itchy palms and itching of eyes, followed by a rash. A dose of 0.0005 gram epinephrine was administered subcutaneously immediately. After the epinephrine injection, an IV rout was established with 0.9 % saline infusion, 0.2 gram of hydrocortisone, and 0.020 gram of chloropiramin was administered intramuscularly. No bronchoobstruction developed. Patient displayed anxiety. After an hour of treatment, the symptoms of anaphylaxis subsided.

## Conclusion

Anaphylaxis can develop as a complication of SIT with any possible manifestation. Treatment of anaphylaxis should start immediately after initial symptoms. The use of epinephrine is recommended.

